# Exploring Parent-Driven Determinants of COVID-19 Vaccination in Indigenous Children: Insights from a National Survey

**DOI:** 10.3390/vaccines13020132

**Published:** 2025-01-28

**Authors:** Abdallah Alami, Sailly Dave, Marwa Ebrahim, Israa Zareef, Caren Uhlik, Julie Laroche

**Affiliations:** Public Health Agency of Canada, Ottawa, ON K1A 0K9, Canada

**Keywords:** Indigenous populations, COVID-19 vaccination, Vaccine hesitancy, Canada, Public health interventions, Pediatric immunization, Indigenous health

## Abstract

**Background:** Globally and in Canada, Indigenous populations have faced heightened vulnerability during pandemics, with historical inequities exacerbated by multigenerational colonial policies. This study aimed to identify parental factors influencing COVID-19 vaccination among Indigenous children in Canada. **Methods:** Data from a nationally representative, cross-sectional survey of parents/guardians with children under 18 years of age were analyzed. The study focused on Indigenous children, examining vaccine uptake, parental hesitancy, and related sociodemographic factors. Multivariable logistic regression models were employed to identify key predictors of COVID-19 vaccination. **Results:** COVID-19 vaccine coverage among Indigenous children was 61.8%, with higher uptake among Inuit (74.4%) children compared to Métis (61.2%) and First Nations (59.6%) children. Nearly half of Indigenous parents (53.4%) expressed hesitancy, primarily due to perceived concerns about insufficient research on the vaccine in children. Higher vaccine uptake was associated with parental education, adherence to routine vaccinations, and urban residence. Conversely, parental hesitancy, particularly related to medical concerns, significantly decreased the likelihood of vaccine uptake. **Conclusions:** The study highlights the complexity of vaccine hesitancy among Indigenous parents. Targeted interventions, including culturally adapted educational initiatives, community engagement, and healthcare provider advocacy, are essential to improve vaccine uptake.

## 1. Introduction

Globally, the historical impact of pandemics on Indigenous populations underscores their heightened vulnerability, a pattern evident in various regions during past outbreaks [1]. Historical data reveal that Indigenous communities experienced significantly higher mortality rates during the 1918 influenza pandemic, as seen with the *Māori* in New Zealand [2] and the *Adivasis* in western India [3]. This pattern persisted during the 2009 H1N1 influenza pandemic, with Indigenous peoples in the Amazon and American Indians/Alaskan Natives in the U.S. facing much higher fatality rates than their non-Indigenous counterparts in both Brazil and U.S. [4,5].

In Canada, the COVID-19 pandemic has disproportionately affected Indigenous populations [6,7]. Data from 2020 and 2021 indicate that COVID-19 mortality rates were higher among First Nations (85.5 deaths per 100,000 population) and Métis (29.4 deaths per 100,000 population) compared to non-Indigenous people (19.1 deaths per 100,000 population) [7]. In addition, the prevalence of non-communicable diseases such as heart diseases, diabetes, and high blood pressure, is higher in Indigenous populations across Canada compared to their non-Indigenous counterparts [8]; 56.7% among First Nations and 58.0% among Métis had three or more comorbidities compared to 46.3% of non-Indigenous people [7]. These underlying health conditions, along with age, race, and ethnicity, contribute to vulnerability to COVID-19 infection and mortality [1].

The health disparities and disproportionate impact of pandemics on Indigenous communities in Canada are deeply rooted in a history of multigenerational colonial policies [9,10]. These policies have unintentionally resulted in significant health inequities and restricted access to equitable and culturally safe healthcare for Indigenous communities [9,11]. Even though more than half of Indigenous peoples currently live in urban areas [12], they continue to experience significant disparities compared to non-Indigenous populations, particularly in remote and rural regions.

The social determinants of health for people in Indigenous communities—characterized by low income, limited access to education, high unemployment, food insecurity, and inadequate sanitation—are critical factors that undermine their overall well-being [13,14]. These challenges are further exacerbated by issues such as overcrowded housing, homelessness, and higher incarceration rates, all of which further compound their vulnerability [10]. As the COVID-19 pandemic unfolded, these pre-existing inequities were further intensified, bringing the vulnerabilities of Indigenous communities to the forefront. The prolonged nature of the pandemic, coupled with the disproportionate impact on Indigenous populations in Canada, highlighted the urgent need for targeted interventions [15]. Public health advisory groups, recognizing the elevated risks, prioritized Indigenous populations for early access to COVID-19 vaccines [16,17]. In response, Indigenous-led immunization clinics were established across several provinces to address barriers to vaccine access and to foster vaccine confidence by providing culturally safe environments [18]. The Canadian Armed Forces also supported vaccination efforts by delivering vaccines to many on-reserve communities [18].

However, ensuring widespread vaccine uptake within these vulnerable populations proved to be a significant challenge. According to the 2023 cycle of the Childhood COVID-19 Immunization Coverage Survey (CCICS), only about two thirds of Indigenous children received at least one dose of the COVID-19 vaccine, despite strong recommendations from public health agencies in Canada [19]. This relatively low vaccine uptake highlights the need to explore the factors influencing parental decisions regarding COVID-19 vaccination for their Indigenous children.

This study seeks to identify the factors driving decisions to vaccinate children against COVID-19 among Indigenous parents in Canada, offering insights that can shape more targeted and effective public health strategies. Understanding these dynamics is essential not only for addressing current gaps in vaccine coverage but also for ensuring that future public health interventions are equitable and responsive to the unique needs of Indigenous populations.

## 2. Materials and Methods

### 2.1. Data Source

This study draws on data from the 2023 CCICS [19], a nationally representative, cross-sectional survey of parents and guardians with children under 18 years of age in Canada. Data collection occurred from 11 April to 26 July 2023 and included responses from parents and guardians of children under 18 years of age across all Canadian provinces and territories. The survey was designed to ensure a balanced representation of males and females.

The 2023 CCICS focused primarily on COVID-19 immunization coverage among children, while also gathering data on seasonal influenza vaccination. The survey enabled an in-depth analysis of socioeconomic and demographic factors influencing vaccination decisions. Additionally, it explored parental intentions regarding children who were eligible for vaccination but had not yet been vaccinated, aiming to identify potential gaps and barriers in vaccination programs. The survey also included questions related to past vaccination history and examined parental knowledge, attitudes, and beliefs (KABs) concerning their child’s COVID-19 and seasonal influenza vaccinations, including vaccine hesitancy.

### 2.2. Study Design

This cross-sectional study employed a probability-based sampling technique targeting a sample population of 11,200 Canadian parents or guardians of children aged 6 months to 17 years [20]. Data collection utilized a multimodal approach, gathering survey responses online and via computer-assisted telephone interviewing (CATI).

Sampling quotas were established for key subpopulations to strengthen statistical power and ensure representativeness at the national level. Also, to ensure that the sampling framework enabled the extrapolation of findings to the broader Canadian population, survey sampling weights were applied. Bootstrap weights were generated and applied to estimate variance, providing robust measures of precision for the survey estimates.

### 2.3. Analytic Sample

This analysis narrows down to a particular subgroup within the overall CCICS, focusing specifically on Indigenous children. While the CCICS captures data from the entire Canadian population, including Indigenous communities, it is not exclusively targeted at them.

To identify the population of interest for this subgroup analysis, we utilized specific survey variables related to racial and ethnic background. We used the variable from the survey question where respondents were asked to self-identify their child’s racial or ethnic background. If respondents indicated that their child was ‘Indigenous (First Nations, Métis, and/or Inuit)’, a subsequent variable captured whether the child identified as First Nations, Métis, or Inuit. All other selections for racial or ethnic background were coded as non-Indigenous. Based on these responses, we classified the child’s identity into the relevant Indigenous subgroups, enabling us to focus the analysis on this population of interest. Throughout this article, we use the term ‘Indigenous’ to refer to people who are First Nations, Inuit, or Métis, unless specified otherwise.

### 2.4. Statistical and Data Analysis

Initial data exploration involved summarizing dependent and independent categorical variables using descriptive statistics. Focusing on Indigenous children presented unique challenges due to minimal sample sizes and infrequent responses in certain categories, necessitating the consolidation of some response options to ensure robust analysis.

We determined COVID-19 vaccine coverage by the receipt of at least one dose of the vaccine. Both unweighted and weighted frequencies and proportions were calculated, stratified by COVID-19 vaccination status, where children were categorized as either vaccinated (having received at least one dose) or unvaccinated (having received no doses). In addition, we conducted sensitivity analyses to explore key variables, comparing Indigenous to non-Indigenous populations, as well as examining differences between urban and rural Indigenous populations. This approach allowed us to identify and understand potential disparities in vaccine uptake within and between these groups.

A Likert plot was used to visually represent parental opinions on various aspects of vaccines, including their safety, their effectiveness, and whether parents felt they had sufficient information to make vaccination decisions for their children. This graphical tool effectively summarized responses, allowing for a quick comparison of viewpoints. To simplify the analysis, we consolidated the original five response categories—‘Strongly agree’, ‘Somewhat agree’, ‘Somewhat disagree’, ‘Strongly disagree’, and ‘Don’t know’—into two categories: agree and disagree. This adjustment was necessary due to the low number of selections for some responses, ensuring interpretable results.

Aligned with our research goals, we examined and included parental hesitancy, along with the reasons behind it, to gain a deeper understanding of the factors influencing COVID-19 vaccination among Indigenous children. The survey asked parents about any hesitancy they had regarding vaccinating their child against COVID-19. For the purpose of this analysis, reasons for hesitancy were categorized into the following:

• Hesitancy stemming from medical concerns: this category included concerns about the child not being at risk of COVID-19 infection, perceived lack of sufficient research on the vaccine in children, the effectiveness and/or safety of the vaccine.
• Hesitancy stemming from trust, information barriers, and personal beliefs: this category encompassed challenges in accessing reliable information sources, difficulties discussing vaccines with healthcare providers, the influence of misinformation, past negative experiences with vaccinations, religious reasons, opposition to vaccines or mandates, other unspecified concerns, and fears related to needles.

Based on these categories, we developed a classification system that grouped respondents into four distinct categories: ‘Not Hesitant’, ‘Hesitant: Medical Concerns’, ‘Hesitant: Trust, Information Barriers, and Personal Beliefs’, and ‘Hesitant: Multiple Reasons’. This classification was instrumental in our regression models, allowing us to effectively analyze the multifaceted hesitancy influences on parental vaccination decisions.

A multivariable logistic regression model was employed to explore factors associated with COVID-19 vaccination among Indigenous children aged 6 months to less than 18 years. To account for the complexities of our survey design, standard errors, coefficients of variation, and confidence intervals were estimated using the bootstrap technique [21]. To address multicollinearity among categorical predictors, those with a Cramer’s V value exceeding 0.4 were flagged for removal [22]. We adopted a ‘best fit’ strategy for constructing multivariable models, initially including all relevant predictors, even those statistically insignificant in the univariate analysis but deemed practically significant. A stepwise backward elimination process was then conducted, guided by the Akaike information criterion (AIC) [23], *p*-values, and adjustments for multiple comparisons. The Archer–Lemeshow goodness of fit test for survey data with logistic regression was used to validate the final model’s fit [24].

R statistical software (version 4·1·3; R Foundation for Statistical Computing, Vienna, Austria) was used for data analysis.

### 2.5. Ethical Considerations

Survey data collection was approved by the Public Health Agency of Canada Research Ethics Board. Participants provided their informed consent to participate in the survey.

## 3. Results

### 3.1. Sociodemographic Characteristics of the Survey Population

Overall, a total of 11,395 Canadian parents/guardians with children under 18 years responded to the survey, a response rate of 29.9% [19]. Within this cohort, the sample size for Indigenous children was 469, representing an overall weighted sample of approximately 227,870 Indigenous children across Canada. The age distribution indicated that 22.5% were aged 6 months to 4 years, 38.4% were between 5 and 11 years, and 39.0% were aged 12 to 17 years. The majority (74.3%) resided in urban settings, while 59.4% of Inuit children lived in Inuit Nunangat. Table 1 provides a detailed breakdown of demographic characteristics, including parental education and household income levels.

### 3.2. COVID-19 Vaccine Coverage

Overall, 61.8% of Indigenous children received at least one dose of a COVID-19 vaccine approved for use in Canada. When disaggregating vaccine coverage by Indigenous identity, vaccine coverage was highest among Inuit children (74.4%), followed by Métis (61.2%) and First Nations (59.6%) children. Coverage varied notably by age group, with children aged 12 to 17 years exhibiting the highest vaccination rate (80.3%), compared to 64% among children aged 5 to 11 years and 26% for those aged 6 months to 4 years. Booster dose uptake was reported for 48.5% of Indigenous children. Detailed coverage rates are presented in Supplementary Table S1.

### 3.3. Seasonal Influenza (Flu) Vaccination and Routine Vaccination Uptake

In examining vaccination patterns prior to the COVID-19 pandemic, we observed a high level of adherence to routine vaccinations among Indigenous children, with 86% of parents reporting that their child had received all recommended routine vaccinations (Table 2). These findings provide an important baseline for understanding how COVID-19 and seasonal influenza (flu) vaccine uptake compare during the pandemic. However, when focusing specifically on seasonal influenza vaccination trends before the pandemic, a different pattern emerges. A significant portion of the surveyed Indigenous population indicated infrequent seasonal influenza (flu) vaccination, with 40.7% of children and 29.6% of parents reporting that they had never received a seasonal influenza (flu) vaccine. This trend may point to broader vaccine hesitancy issues that extend beyond COVID-19 vaccines. For the 2022–2023 flu season, the majority of parents (63.4%) and their children (67.5%) did not receive the seasonal flu vaccine, suggesting a continued pattern of seasonal flu vaccine avoidance during this period.

### 3.4. Parental Attitudes, Influences, and Barriers to COVID-19 Vaccination

Among parents of unvaccinated Indigenous children, 81.6% indicated they are unlikely (‘Probably won’t’ or ‘Definitely won’t’) to vaccinate their child against COVID-19 in the future. In addition, hesitancy, expressed by 46.6% of parents, was predominantly due to perceived insufficient research on the vaccine in children (79.9%) followed by concerns about vaccine safety and potential side effects (67.2%), lack of confidence in vaccine effectiveness (53.5%), and believing that the child was not at risk of contracting COVID-19 (14.9%). Healthcare providers were the most favored as a trusted source for vaccine information, trusted by 40.8% of parents of Indigenous children, followed by scientific publications/journals and/or international organizations such as the World Health Organization (WHO) (22.4%), and the Public Health Agency of Canada/Health Canada (17.2%). Table 3 outlines key parental attitudes, hesitancy, and influences.

**Figure 1:**
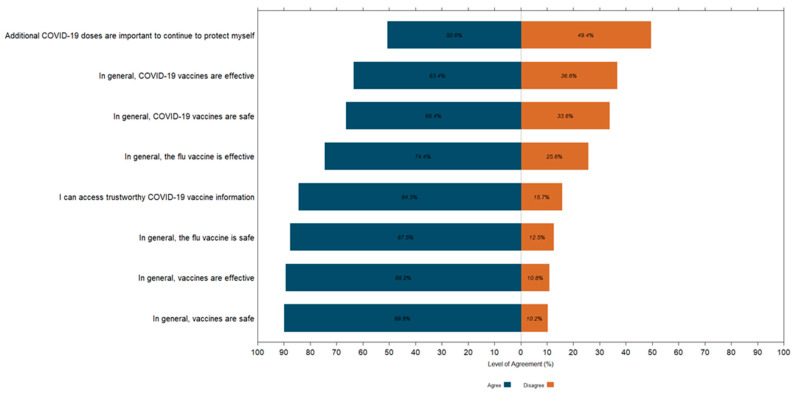
Parental Attitudes Toward Vaccine Safety and Effectiveness: Likert Plot Distribution of Responses on Vaccine Belief Statements.

Regarding overall vaccine beliefs, a substantial majority of parents of Indigenous children (89.9%) agreed that vaccines are generally safe, and 89.2% believed in their effectiveness (Figure 1). However, confidence in the COVID-19 vaccine was lower, with 64.4% of parents considering it safe and 63.4% believing it effective. Opinions were divided on the need for additional COVID-19 doses, with 50.6% in agreement and 49.4% in disagreement. Despite these mixed views, 84.3% of parents felt they had access to enough trustworthy information to make an informed decision about COVID-19 vaccination.

### 3.5. Urban Indigenous Compared to Rural Indigenous

The comparison between urban and rural Indigenous populations revealed notable differences in COVID-19 vaccine coverage and related vaccination behaviors. Urban Indigenous children had a higher COVID-19 vaccine uptake, with 64.9% having received at least one dose, compared to 56.7% of rural Indigenous children. However, the uptake of recommended routine vaccinations and seasonal flu vaccine uptake during the current season were similar across both groups, as were future parental intentions regarding COVID-19 and seasonal flu vaccinations. Notably, rural Indigenous parents exhibited slightly higher hesitancy towards vaccinating their children against COVID-19 compared to urban Indigenous parents (Supplementary Table S2).

### 3.6. Indigenous Compared to Non-Indigenous

Similarly, as part of our sensitivity analysis, a comparison between Indigenous and non-Indigenous populations revealed that Indigenous parents were more hesitant to vaccinate their children against COVID-19, with 53.4% reporting hesitancy compared to 43.5% of non-Indigenous parents. The uptake of recommended routine vaccinations was also slightly lower among Indigenous children (86.1%) compared to non-Indigenous children (89%). Despite these differences, future parental intentions regarding COVID-19 and flu vaccinations were similar between the two groups (Supplementary Table S3).

### 3.7. Determinants of COVID-19 Vaccination

As outlined in Table 4, our multivariable logistic regression models identified several key predictors of COVID-19 vaccination among Indigenous children. Age was a particularly influential factor, with younger children (aged 6 months to 4 years) being significantly less likely to receive the vaccine compared to older children (aged 12 to 17 years), as reflected by an aOR of 0.03 (95% CI: 0.02–0.03, *p* < 0.001). Similarly, children aged 5 to less than 12 years also demonstrated reduced odds of vaccination. Sex differences were also evident, with male children more likely to be vaccinated than females (aOR: 1.21, 95% CI: 1.11–1.32, *p* < 0.001). Additionally, children living in urban areas had higher odds of vaccination compared to those in rural settings (aOR: 1.58, 95% CI: 1.36–1.83, *p* < 0.001).

Parental educational attainment emerged as another significant determinant. Children whose parents had postsecondary education below a bachelor’s degree (aOR: 1.21, 95% CI: 1.01–1.46, *p* = 0.042) and those with a bachelor’s degree or higher (aOR: 1.24, 95% CI: 1.06–1.46, *p* = 0.009) were more likely to be vaccinated compared to children of parents with a high school education or less. Furthermore, adherence to routine childhood vaccination schedules strongly predicted COVID-19 vaccination, with children who had received all recommended routine vaccinations being significantly more likely to receive the COVID-19 vaccine (aOR: 2.43, 95% CI: 2.25–2.61, *p* < 0.001). Household income was another significant determinant. Children from higher-income households ($80,000–$149,999: aOR: 2.00, 95% CI: 1.76–2.29, *p* < 0.001; $150,000 and above+: aOR: 1.48, 95% CI: 1.21–1.80, *p* < 0.001) had greater odds of being vaccinated compared to those from households earning under $40,000.

Parental hesitancy significantly decreased the likelihood of a child receiving the COVID-19 vaccine. Hesitancy due to medical concerns had the most substantial negative impact, reducing the likelihood of vaccination by 94% (aOR: 0.06, 95% CI: 0.04–0.07, *p* < 0.001). Similarly, hesitancy related to trust, information barriers, and personal beliefs also significantly decreased the odds of vaccination (aOR: 0.08, 95% CI: 0.07–0.10, *p* < 0.001).

## 4. Discussion

The findings of this study provide critical insights into the factors influencing COVID-19 vaccine uptake among Indigenous children in Canada. Vaccine coverage within this population was 61.8%, with hesitancy reported by nearly half of the parents, primarily due to concerns about insufficient research on vaccines in children. Parental education, household income, and adherence to routine vaccinations emerged as significant predictors of vaccine uptake, while parental hesitancy, particularly driven by medical concern, was strongly linked to higher odds of non-vaccination. These findings emphasize the complex interplay of factors influencing vaccine uptake, underscoring the need to better understand the disparities observed among Indigenous and non-Indigenous populations. As previously mentioned, our results show that COVID-19 vaccine coverage was 61.8% in Indigenous children, while vaccine coverage in non-Indigenous children was 67.3%. These findings align with previous studies in Canada that have documented racial disparities in COVID-19 vaccination, showing lower vaccine uptake among Indigenous populations, including First Nations, Inuit, and Métis, compared to the general population [9,25]. Similar trends have been observed globally. A recent study in Brazil revealed significant heterogeneities in vaccination coverage across Indigenous districts, with overall lower coverage among Indigenous peoples compared to non-Indigenous populations [26]. This was attributed to the absence of a coordinated strategy to reinforce the importance of vaccination, ensure access to trustworthy information, and provide necessary resources in extreme situations [26]. In the United States, while urban American Indian and Alaskan Native (AI/AN) parents initially vaccinated their children against COVID-19 at rates consistent with other US populations, over time, vaccination and booster rates among AI/AN children have lagged behind those of other racial and ethnic minority groups [27]. Additionally, while higher education is generally less prevalent among AI/AN populations and typically associated with higher vaccination rates, it can paradoxically lead to lower child vaccination rates in contexts where there is strong distrust of the vaccine [27].

The findings from this analysis also highlight the complexity and multifaceted nature of Indigenous parental hesitancy toward vaccinating their children against COVID-19. A recent review on COVID-19 vaccine hesitancy among marginalized populations, including American Indians, Native Hawaiians, and Indigenous peoples in the United States and Canada identified multiple and complex drivers of hesitancy [28]. Structural barriers, such as institutional mistrust and a desire for autonomy, were significant factors contributing to this hesitancy [28]. In Canada, research on disparities in vaccination uptake among racialized and Indigenous peoples tend to be evaluated through the lens of vaccine reluctance [29]. While some evidence points to COVID-19 vaccine hesitancy in these communities, a recent Canadian study found that many participants expressed willingness to vaccinate but faced substantial social and structural barriers [29]. Historical context is also pivotal in understanding this reluctance. Due to past involuntary medical treatments and experimentation, many Indigenous peoples may be reluctant to receive vaccines. For example, Mosby and Swidrovich (2021) reported that some First Nations, Métis, and Inuit elders in Canada recall vaccines being tested on them as children in residential schools and are wary of the new vaccines [10]. Adding to this concern, the federal government’s prioritization of Indigenous populations for COVID-19 vaccinations was met with suspicion by some, who had concerns that they were again being used to test new vaccines [10].

The importance of culturally appropriate healthcare is a recurring theme for Indigenous peoples, not only in the context of COVID-19 vaccines but also for non-COVID vaccines and healthcare more broadly [29]. This emphasizes the need for healthcare practices that respect and integrate Indigenous cultural values and traditions. Further research is essential to understand the structural and systemic factors contributing to hesitancy and vaccine uptake among First Nations, Métis, and Inuit populations. In light of our findings, several strategic imperatives can be suggested to enhance vaccine uptake [30,31]:

• **Continue and expand on culturally adapted educational initiatives:** Improving COVID-19 vaccine uptake requires the development of culturally responsive educational resources. Collaborating with Indigenous community leaders and health experts to create and disseminate these materials can help bridge gaps in understanding and trust, particularly where educational disparities exist.
• **Continue to improve community-centric approaches:** Addressing COVID-19 vaccine hesitancy and low intention to vaccinate within Indigenous populations necessitates a focus on community engagement. Initiatives should prioritize transparent, dialogue-driven awareness campaigns that are tailored to specific community concerns and historical contexts. This approach can foster greater confidence in COVID-19 vaccination efforts.
• **Strengthening healthcare provider (HCP)-led advocacy:** Given that healthcare providers are the most trusted source of vaccine information; it is crucial to support and enable them to play a central role in immunization advocacy. Providing HCPs with training and resources to effectively communicate vaccine benefits, address concerns, and deliver culturally competent care can significantly increase vaccine uptake among Indigenous populations.

While these recommendations are tailored to address determinants to COVID-19 vaccination identified in this study, they can also inform strategies for the introduction of future vaccines within Indigenous communities. Unlike routine childhood vaccinations, which generally see higher vaccine acceptance and coverage, new vaccines may face additional challenges that require targeted, culturally responsive strategies to ensure equitable and inclusive vaccination efforts.

This study is the first to assess factors associated with COVID-19 vaccination among Indigenous children in Canada, using a nationally representative sample and contributing valuable insights to the existing research on vaccine acceptance and hesitancy. A major strength of this study is its large sample size, which allows for detailed analysis across various sociodemographic variables. The population-based design and use of survey weights ensure that the findings are nationally representative, enabling broader inferences about the Canadian population. Additionally, the study provides a comprehensive examination of sociodemographic factors, routine vaccination adherence, and parental knowledge, attitudes, and beliefs within the context of the COVID-19 pandemic. Several limitations must also be considered to contextualize the findings. While this survey captures Indigenous perspectives, it was not specifically designed to assess the Indigenous population exclusively. Additionally, inherent challenges in surveying populations on-reserve may affect the representativeness of the data for these communities. The study’s methodology, which involved surveying only one adult (parent/guardian) about one randomly selected child, does not account for the vaccination status of other siblings within the family. Furthermore, the survey was limited to English and French, potentially hindering participation from Indigenous communities where these are not the primary languages spoken. Finally, the reliance on self-reported information introduces the possibility of recall bias, a common limitation in population surveys.

## 5. Conclusions

This study provides a comprehensive analysis of the factors influencing COVID-19 vaccination among Indigenous children in Canada, revealing significant disparities in vaccine uptake driven by parental hesitancy, educational attainment, and adherence to routine vaccination schedules. Despite the general acceptance of vaccines, specific concerns about the COVID-19 vaccine persists. Addressing these concerns requires a multifaceted approach, including the development of culturally adapted educational initiatives, community-centric engagement strategies, and strengthened advocacy from healthcare providers. Further research is essential to continue exploring these dynamics and to develop interventions that are both culturally respectful and responsive to the needs of Indigenous peoples.

## Figures and Tables

**Table 1:** Characteristics of Survey Population.

	Overall N (Weighted %)	Vaccinated Children N (Weighted %)	Unvaccinated Children N (Weighted %)
**Sample size**	469	303 (61.8)	166 (38.2)
**Child’s Indigenous Identity ^1^**			
First Nations	208 (39.4)	130 (38.0)	78 (41.7)
Métis	139 (36.0)	93 (36.0)	54 (36.1)
Inuit	53 (6.9)	42 (9.1)	17 (5.0)
Prefer not to answer/Don’t know	69 (17.6)	45 (17.6)	24 (17.7)
**Child age**			
6 months and less than 5 years	103 (22.5)	27 (9.5)	76 (43.6)
5–11 years	166 (38.4)	110 (39.8)	56 (36.3)
12–17 years	200 (39.0)	166 (50.7)	34 (20.1)
**Age of responding parent**			
18–39	163 (33.0)	77 (22.1)	86 (50.8)
40–49	179 (45.1)	134 (53.3)	45 (31.6)
50+	103 (21.9)	77 (24.6)	26 (17.5)
**Child sex at birth**			
Male	231 (47.7)	151 (46.2)	80 (50.0)
Female	238 (52.3)	152 (53.8)	86 (50.0)
**Sex of responding parent**			
Male	166 (36.4)	97 (31.6)	69 (44.3)
Female	298 (63.6)	204 (68.4)	94 (55.7)
**Urban/Rural setting**			
Urban	311 (74.3)	205 (76.8)	106 (70.2)
Rural	152 (25.7)	98 (23.2)	54 (29.8)
**Primary residence on reserve ^2^**			
Yes	16 (6.8)	10 (3.8)	6 (11.2)
No	191 (93.2)	119 (96.2)	72 (88.8)
**Primary residence in Inuit Nunangat (Inuvialuit, Nunavik, Nunatsiavut, or Nunavut) ^3^**			
Yes	32 (59.4)	22 (57.1)	10 (66.5)
No	25 (40.6)	19 (42.9)	6 (33.5)
**Education of responding parent**			
High school or less than high school	110 (18.3)	71 (19.1)	39 (17.1)
Postsecondary below bachelor’s	189 (43.1)	112 (39.2)	77 (49.3)
Bachelor’s or above	161 (38.6)	114 (41.7)	47 (33.6)
**Working/volunteer sector of responding parent ^4^**			
High-risk sector	183 (39.7)	130 (43.8)	53 (32.8)
Non-high-risk sector	270 (60.3)	168 (56.2)	102 (67.2)
**Total household income (CAD $)**			
Under $40,000	82 (17.2)	48 (15.2)	34 (20.5)
$40,000–$79,999	86 (19.2)	51 (18.2)	35 (21.0)
$80,000–$149,999	159 (38.2)	105 (40.4)	54 (34.6)
$150,000 and above	113 (25.3)	80 (26.2)	33 (24.0)
**Existing medical condition of child ^5^**			
With medical condition	74 (17.1)	43 (15.2)	31 (20.2)
Without medical condition	385 (82.9)	255 (84.8)	130 (79.8)
**Existing child disability ^6^**			
With disability	51 (12.7)	31 (11.8)	20 (14.2)
Without disability	410 (87.3)	267 (88.2)	143 (85.8)

^1^ More than one response option could be selected. ^2^ Among children who identify as First Nation. ^3^ Among children who identify as Inuit. ^4^ Currently working or volunteering in high-risk sectors such as healthcare, laboratory services, childcare, schools, occupations involving animal exposure, emergency services, or other high-risk roles (e.g., correctional facility staff, crew members on ships or aircraft, military personnel, humanitarian relief workers, or essential community service providers). ^5^ Medical conditions of interest are based on the Canadian Immunization Guide and include sickle cell anemia or thalassemia major; neurologic or neurodevelopmental disorders; asthma or other chronic lung diseases; chronic liver, heart or kidney disease; diabetes; obesity or Down Syndrome; immune suppression (chemotherapy, radiotherapy, steroid use, HIV, organ transplant) or cancer and other medical conditions. ^6^ A person who has a long-term or recurring impairment (such as vision-, hearing-, mobility-, flexibility-, dexterity-, pain-, learning-, developmental-, memory-, or mental health-related) which limits their daily activities inside or outside the home (such as at school, work, or in the community in general).

**Table 2:** Vaccination Uptake and History Among Indigenous Children and Responding Parents.

	**N (Weighted %)**
**Uptake of recommended routine child vaccination**	
Yes, all routine vaccinations	409 (86.0)
None or only some were received	55 (14.0)
**Child frequency of receiving a flu vaccine prior to the COVID-19 pandemic**	
Every flu season	99 (21.4)
Most flu seasons	66 (15.4)
Some flu seasons (including once only)	118 (22.3)
Never	177 (40.8)
**Child received flu vaccine during the 2022–2023 season**	
Yes, child received the flu vaccine	150 (32.5)
No, child did not receive the flu vaccine	307 (67.5)
**Did child receive a COVID-19 booster dose?**	
Yes, child received a booster	130 (48.5)
No, child did not receive a booster	137 (51.5)
**Child ever been diagnosed with COVID-19**	
Yes, diagnosed with COVID-19	251 (49.6)
No, has not received a COVID-19 diagnosis	203 (46.4)
Don’t know	14 (4.0)
**Responding parent frequency of receiving a flu vaccine prior to the COVID-19 pandemic**	
Every flu season	85 (17.8)
Most flu seasons	101 (21.9)
Some flu seasons (including once only)	152 (30.8)
Never	128 (29.6)
**Responding parent flu vaccination during the 2022–2023 flu season**	
Yes, vaccinated	168 (36.6)
No, did not receive the vaccine	297 (63.4)
**Responding parent COVID-19 vaccination**	
Yes, received at least 1 dose of COVID-19 vaccine	411 (86.1)
No, did not receive the COVID-19 vaccine	55 (13.9)

**Table 3:** Parental Attitudes, Hesitancy, and Influences on Child COVID-19 Vaccination.

	**N (Weighted %)**
**How likely is it that you will have your child vaccinated against COVID-19? ^1^**	
Definitely will/Probably will	32 (18.4)
Probably won’t/Definitely won’t	121 (81.6)
**In the future, how likely is it that your child will receive a COVID-19 booster dose?**	
Definitely will/Probably will	44 (32.9)
Probably won’t/Definitely won’t	84 (67.1)
**How likely is it that you will have your child vaccinated against the flu in the next flu season?**	
Definitely will/Probably will	240 (54.4)
Probably won’t/Definitely won’t	194 (45.6)
**Were you hesitant to vaccinate your child against the flu during this flu season?**	
Yes	150 (35.1)
No	301 (64.9)
**Did you refuse to get the flu vaccine for your child during this flu season?**	
Yes	116 (40.4)
No	191 (59.6)
**Impact of COVID-19 pandemic on parental decision to vaccinate children against flu**	
Yes, it motivated me to getmy child vaccinated against the flu	46 (10.4)
Yes, it made me not want to getmy child vaccinated against the flu	49 (11.1)
No, it did not impact my decision one way or another	361 (78.5)
**Were you hesitant to vaccinate your child against COVID-19? ^2^**	
Hesitant	230 (53.4)
Not hesitant	227 (46.6)
**Reasons for hesitancy to vaccinate child against COVID-19? ^3^**	
My child fears needles	20 (6.5)
My child is not at risk of contracting COVID-19	32 (14.9)
I wanted to first discuss the flu vaccine with my child’s health care practitioner	32 (10.7)
I have concerns that not enough research on the vaccine has been carried out in children	189 (79.9)
I was concerned about the effectiveness of the COVID vaccine	130 (53.5)
I had concerns about the safety of the COVID vaccine and/or side effects	162 (67.2)
My child had a bad experience with previous vaccines	15 (5.8)
I did not know where to obtain reliable information	36 (12.0)
Religious or philosophical reasons	19 (8.4)
Misinformation	14 (6.7)
Against vaccination or mandate	1 (0.6)
Other	15 (7.2)
**Most trusted source of information about COVID-19 vaccines**	
Health care providers	171 (40.8)
Family/friends	16 (3.0)
Local public health unit/clinic	40 (6.3)
Public Health Agency of Canada/Health Canada	85 (17.2)
News and social media (e.g., Twitter, Facebook)	13 (2.9)
Scientific publications/journals or international sources (WHO)	85 (22.4)
Other sources	31 (7.4)
**Parental Opinion on Safety of Vaccines**	
Agree	408 (89.8)
Disagree	47 (10.2)
**Parental Opinion on Effectiveness of Vaccines**	
Agree	411 (89.2)
Disagree	46 (10.8)
**Parental Opinion on Safety of Flu Vaccines**	
Agree	380 (87.5)
Disagree	54 (12.5)
**Parental Opinion on Effectiveness of Flu Vaccines**	
Agree	330 (74.4)
Disagree	107 (25.6)
**Parental Opinion on Safety of COVID Vaccines**	
Agree	302 (66.4)
Disagree	133 (33.6)
**Parental Opinion on Effectiveness of COVID Vaccines**	
Agree	293 (63.4)
Disagree	149 (36.6)
**Parental Opinion on Additional Doses of COVID Vaccines**	
Agree	235 (50.6)
Disagree	205 (49.4)
**Parental Opinion on Access to Enough Trustworthy Information About COVID-19 Vaccines to Make an Informed Decision**	
Agree	390 (84.3)
Disagree	65 (15.7)

^1^ Among parents of unvaccinated Indigenous children. ^2^ Vaccine hesitancy refers to a delay in acceptance or refusal of vaccines despite availability. ^3^ More than one response option could be selected.

**Table 4:** Key Predictors of COVID-19 Vaccination Among Indigenous Children: Results from Multivariable Logistic Regression Model.

Characteristic	aOR ^1^	95% CI ^2^	*p*-Value
**Age of Child**			<0.001
12 years to less than 18 years	—	—	
6 months–4 years	0.03	0.02, 0.03	<0.001
5 years to less than 12 years	0.47	0.43, 0.52	<0.001
**Child Sex**			<0.001
Female	—	—	
Male	1.21	1.11, 1.32	<0.001
**Residential setting**			<0.001
Rural	—	—	
Urban	1.58	1.36, 1.83	<0.001
**Parental educational attainment**			0.030
High school or less than high school	—	—	
Postsecondary below bachelor’s	1.21	1.01, 1.46	0.042
Bachelor’s or above	1.24	1.06, 1.46	0.009
**Child routine vaccination**			<0.001
None or only some were received	—	—	
Yes, all routine vaccinations	2.43	2.25, 2.61	<0.001
**Household Income**			<0.001
Under $40,000	—	—	
$40,000–$79,999	1.17	1.06, 1.29	0.003
$80,000–$149,999	2.00	1.76, 2.29	<0.001
$150,000 and above	1.48	1.21, 1.80	<0.001
**Hesitancy**			<0.001
Not hesitant	—	—	
Hesitant: Multiple reasons	0.04	0.03, 0.06	<0.001
Hesitant: Medical concerns	0.06	0.04, 0.07	<0.001
Hesitant: Trust, information barriers, and personal beliefs	0.08	0.07, 0.10	<0.001

^1^ aOR = adjusted odds ratio, ^2^ CI = confidence interval.

## Data Availability

Detailed results tables and the methodological report can be accessed through the Library and Archives Canada website. Survey results can be accessed at: https://www.canada.ca/en/public-health/services/immunization-vaccines/vaccination-coverage/childhood-covid-19-immunization-coverage-survey-2023-results.html (accessed on 8 December 2024).

## Supplementary Materials

The following supporting information can be downloaded at: https://www.mdpi.com/article/10.3390/vaccines13020132/s1. Table S1: Coverage Stratified by Demographic Factors; Table S2: Urban vs. Rural Indigenous; Table S3: Indigenous vs. Non-Indigenous.
