# Supplementary material for: Exploring Parent-Driven Determinants of COVID-19 Vaccination in Indigenous Children: Insights from a National Survey
Source: Vaccines (Basel). 2025 Jan 28;13(2):132. doi: 10.3390/vaccines13020132 (PMC11860257; doi:10.3390/vaccines13020132)
Supplement: Supplementary file 1 [file vaccines-13-00132-s001.zip › vaccines-3390274-supplementary_Final.pdf]

Supplementary Material

Exploring Parent-Driven Determinants of COVID-19 Vaccination in Indigenous Children: Insights from a National Survey

Abdallah Alami, Sailly Dave, Marwa Ebrahim, Israa Zareef, Caren Uhlik, Julie Laroche

S1. Coverage Stratified by Demographic Factors

	N (weighted %)	N (weighted %)
**Child age**		
6 months - 4 years	27 (26.0)	76 (74.0)
5 - 11 years	110 (64.0	56 (36.0)
12 - 17 years	166 (80.3)	34 (19.7)
**Residential setting**		
Urban	205 (64.9)	106 (35.1)
Rural	98 (56.7)	54 (43.3)
**Indigenous identity[1]**		
First Nations	130 (59.6)	78 (40.4)
Métis	93 (61.2)	54 (38.8)
Inuk/Inuit	42 (74.7)	17 (25.3)
**Parental educational attainment**		
High school or Less than high school	71 (64.5)	39 (35.5)
Postsecondary below Bachelors	112 (56.4)	77 (43.6)
Bachelors or above	114 (66.8)	47 (33.2)
**Child with medical condition[2]**		
Chronic medical condition	43 (55.6)	31 (44.4)

No chronic medical condition	255 (63.8)	130 (36.2)
**Child with disability[3]**		
Yes, child identify as a person with a disability	31 (57.4)	20 (42.6)
No child does not identify as a person with a disability	267 (62.5)	143 (37.5)
**Parent working sector[4]**		
Yes, I work/volunteer in a higher risk sector	130 (69.4)	53 (30.6)
No, I work/volunteer in a higher risk sector	168 (58.7)	102 (41.3)
**Household income (CAD $)**		
Less than $40,000	48 (54.5)	34 (45.5)
$40,000–$79,999	51 (58.2)	35 (41.8)
$80,000–$149,999	105 (65.3)	54 (34.7)
$150,000 and above	80 (63.7)	33 (36.3)

[1] More than one response option could be selected.

[2]Medical conditions of interest are based on the Canadian Immunization Guide and include sickle cell anemia or thalassemia major, neurologic or neurodevelopmental disorders, asthma or other chronic lung diseases, chronic liver, heart or kidney disease, diabetes, obesity or Down Syndrome, immune suppression (chemotherapy, radiotherapy, steroid use, HIV, organ transplant) or cancer and other medical conditions.

[3]A person who has a long-term or recurring impairment (such as vision, hearing, mobility, flexibility, dexterity, pain, learning, developmental, memory or mental health-related) which limits their daily activities inside or outside the home (such as at school, work, or in the community in general).

[4]Currently working or volunteering in high-risk sectors such as healthcare, laboratory services, childcare, schools, occupations involving animal exposure, emergency services, or other high-risk roles (e.g., correctional facility staff, crew members on ships or aircraft, military personnel, humanitarian relief workers, or essential community service providers).

S2. Urban vs Rural indigenous

	Urban Indigenous N (weighted %)	Rural Indigenous N (weighted %)
**Child COVID-19 vaccine coverage**		
Yes, child received at least 1 dose of COVID-19 vaccine	205 (64.9)	98 (56.7)
No, child did not receive the COVID-19 vaccine	106 (35.1)	54 (43.3)
**Uptake of recommended routine child vaccination**		
Yes, all routine vaccinations	281 (85.9)	139 (86.9)
None or only some were received	41 (14.1)	15 (13.1)
**Child frequency of receiving a flu vaccine prior to the COVID-19 pandemic**		
Every flu season	69 (21.9)	30 (19.8)
Most flu seasons	52 (16.6)	17 (12.9)
Some flu seasons (including once only)	72 (21.6)	48 (26.1)
Never	121 (39.9)	55 (41.2)
**Child received flu vaccine during this season**		
Yes, child got flu vax	103 (32.5)	48 (32.4)
No, child did not get the flu vax	209 (67.5)	100 (67.6)
**Child ever been diagnosed with COVID-19**		
Yes, diagnosed with COVID	166 (49.6)	87 (48.9)
No, did not get COVID-19 diagnosis	149 (47.2)	63 (43.6)
Don't know	8 (3.2)	7 (7.5)
**Did child receive a COVID-19 booster dose?**[1]		
Yes, child received a booster	88 (50.1)	42 (43.0)
No, child did not receive a booster	92 (49.9)	45 (57.0)

**Were you hesitant to vaccinate your child against COVID-19[1]**		
Hesitant	153 (51.6)	78 (57.1)
Not hesitant	159 (48.4)	72 (42.9)
**Were you hesitant to vaccinate your child against the flu, during this flu season?**		
Yes	97 (33.1)	51 (38.2)
No	210 (66.9)	96 (61.8)
**In the future, how likely is it that child will get a COVID-19 booster dose?**		
Definitely will/ Probably will	27 (34.0)	17 (30.3)
Probably won't/ Definitely won't	55 (66.0)	29 (69.7)
**How likely is it that you will get your child vaccinated against the flu in the next flu season?**		
Definitely will/ Probably will	167 (55.3)	76 (51.9)
Probably won't/ Definitely won't	129 (44.7)	68 (48.1)
**How likely is it that you will get your child vaccinated against the COVID in the future?**		
Definitely will/ Probably will	23 (18.8)	11 (19.1)
Probably won't/ Definitely won't	81 (81.2)	42 (80.9)
**Responding parent COVID-19 vaccination**		
Yes, Received at least 1 dose of COVID-19 vaccine	288 (88.1)	135 (83)
No, Did not receive the COVID-19 vaccine	34 (11.9)	21 (17.0)
**Impact of COVID-19 Pandemic on Parental Decision to Vaccinate Children Against Flu**		
Yes, it motivated me to get my child vaccinated against the flu	34 (11.7)	13 (6.8)
Yes, it made me not want to get my child vaccinated against the flu	28 (9.1)	19 (13.5)

No, it did not impact my decision one way or another	249 (79.1)	119 (79.7)

[1] *Vaccine hesitancy refers to a delay in acceptance or refusal of vaccines despite availability.*

S3. Indigenous vs non-Indigenous

	Indigenous N (weighted %)	Non-indigenous N (weighted %)
**Child COVID-19 vaccine coverage**		
No, child did not receive the COVID-19 vaccine	166 (38.2)	3400 (32.7)
Yes, child received at least 1 dose of COVID-19 vaccine	303 (61.8)	7159 (67.3)
**Uptake of recommended routine child vaccination**		
Yes, all routine vaccinations	425 (86.1)	9667 (89.0)
None or only some were received	57 (13.9)	1132 (11.0)
**Child frequency of receiving a flu vaccine prior to the COVID-19 pandemic**		
Every flu season	100 (21.3)	2702 (25.3)
Most flu seasons	69 (15.4)	1501 (14.1)
Some flu seasons (including once only)	121 (22.6)	2030 (19.2)
Never	180 (40.7)	4075 (41.5)
**Child received flu vaccine during this season**		
Yes, child got flu vaccine	153 (32.3)	3780 (34.5)
No, child did not get the flu vaccine	313 (67.7)	6691 (65.5)
**Child ever been diagnosed with COVID-19**		
Yes, diagnosed with COVID	254 (48.8)	5726 (52.2)
No, did not get COVID-19 diagnosis	217 (47.0)	4896 (45.6)
Don’t know	15 (4.2)	221 (2.2)
**Did child receive a COVID-19 booster dose?**[1]		
Yes, child received a booster	130 (48.5)	3098 (48.6)
No, child did not receive a booster	137 (51.5)	3068 (51.4)
**Were you hesitant to vaccinate your child against COVID-19**[1]		

Hesitant	234 (53.4)	4479 (43.5)
Not hesitant	233 (46.6)	5973 (56.5)
**Were you hesitant to vaccinate your child against the flu, during this flu season**		
Yes	151 (34.9)	2827 (28.8)
No	309 (65.1)	7437 (71.2)
**In the future, how likely is it that child will get a COVID-19 booster dose?**		
Definitely will/ Probably will	44 (32.9)	1023 (38.1)
Probably won't/ Definitely won't	84 (67.1)	1654 (61.9)
**How likely is it that you will get your child vaccinated against the flu in the next flu season**		
Definitely will/ Probably will	246 (54.0)	5598 (54.4)
Probably won't/ Definitely won't	200 (46.0)	4313 (45.6)
**How likely is it that you will get your child vaccinated against the COVID in the future?**		
Definitely will/ Probably will	35 (18.5)	633 (17.5)
Probably won't/ Definitely won't	128 (81.5)	2553 (82.5)
**Responding parent COVID vaccination**		
Yes, received at least 1 dose of COVID-19 vaccine	426 (86.3)	9931 (91.9)
No, did not receive the COVID-19 vaccine	57 (13.7)	822 (8.1)
**Impact of COVID-19 Pandemic on Parental Decision to Vaccinate Children Against Flu**		
Yes, it motivated me to get my child vaccinated against the flu	47 (10.3)	1195 (11.7)
Yes, it made me not want to get my child vaccinated against the flu	50 (11.1)	868 (8.3)
No, it did not impact my decision one way or another	370 (78.5)	8392 (80)

[1] *Vaccine hesitancy refers to a delay in acceptance or refusal of vaccines despite availability.*
